# 2,4-Dichlorophenoxyacetic acid promotes *S*-nitrosylation and oxidation of actin affecting cytoskeleton and peroxisomal dynamics

**DOI:** 10.1093/jxb/eru237

**Published:** 2014-06-09

**Authors:** M. Rodríguez-Serrano, D. M. Pazmiño, I. Sparkes, A. Rochetti, C. Hawes, M. C. Romero-Puertas, L. M. Sandalio

**Affiliations:** ^1^Departamento de Bioquímica, Biología Celular y Molecular de Plantas, Estación Experimental del Zaidín, CSIC, Apartado 419, 18080 Granada, Spain; ^2^School of Biological & Medical Sciences, Oxford Brookes University, Oxford OX3 0BP, UK

**Keywords:** Actin, cytoskeleton, 2, 4-D, nitric oxide, peroxisomes, ROS, S-nitrosylation, xanthine dehydrogenase.

## Abstract

2,4-Dichlorophenoxyacetic acid (2,4-D) is a synthetic auxin used as a herbicide to control weeds in agriculture. A high concentration of 2,4-D promotes leaf epinasty and cell death. In this work, the molecular mechanisms involved in the toxicity of this herbicide are studied by analysing in *Arabidopsis* plants the accumulation of reactive oxygen species (ROS) and nitric oxide (NO), and their effect on cytoskeleton structure and peroxisome dynamics. 2,4-D (23mM) promotes leaf epinasty, whereas this process was prevented by EDTA, which can reduce ·OH accumulation. The analysis of ROS accumulation by confocal microscopy showed a 2,4-D-dependent increase in both H_2_O_2_ and O_2_·^–^, whereas total NO was not affected by the treatment. The herbicide promotes disturbances on the actin cytoskeleton structure as a result of post-translational modification of actin by oxidation and *S-*nitrosylation, which could disturb actin polymerization, as suggested by the reduction of the F-actin/G-actin ratio. These effects were reduced by EDTA, and the reduction of ROS production in *Arabidopsis* mutants deficient in xanthine dehydrogenase (*Atxdh*) gave rise to a reduction in actin oxidation. Also, 2,4-D alters the dynamics of the peroxisome, slowing the speed and shortening the distances by which these organelles are displaced. It is concluded that 2,4-D promotes oxidative and nitrosative stress, causing disturbances in the actin cytoskeleton, thereby affecting the dynamics of peroxisomes and some other organelles such as the mitochondria, with xanthine dehydrogenase being involved in ROS production under these conditions. These structural changes in turn appear to be responsible for the leaf epinasty.

## Summary

2,4-D affects actin polymerization by post-translational modifications (carbonylation and *S*-nitrosylation), thereby disturbing the actin cytoskeleton and the dynamics of peroxisomes. Xanthine dehydrogenase is involved in ROS production under these conditions.

## Introduction

Auxin herbicides have been one of the most successful chemicals used to control weeds in agriculture. 2,4-Dichlorophenoxyacetic acid (2,4-D) was the first synthetic auxin analogue of indole-3-acetic acid (IAA, natural auxin) used in agriculture (Grossmann, 2010). The dose-dependent mode of action of 2,4-D causes different effects on sensitive species, and this marks the difference between its action as a growth promoter or as a herbicide. Thus, at low concentrations, 2,4-D stimulates growth and developmental processes, but at high concentrations upsets normal growth and provokes lethal damage in the plant (Grossmann, 2010). Common visible effects induced by 2,4-D and auxin herbicides are epinastic deformations, stem curvature, senescence, and growth inhibition of roots and shoots (Grossmann *et al.*, 2001; Pazmiño *et al.*, 2012). Pea plants exposed to 2,4-D also develop oxidative stress symptoms characterized by H_2_O_2_ overaccumulation, lipid peroxidation, protein oxidation, and the induction of proteolysis (Romero-Puertas *et al.*, 2004*a*; Pazmiño *et al.*, 2011, 2012). In young leaves, reactive oxygen species (ROS) accumulation is involved in 2,4-D-induced epinasty, while in adult leaves ROS overproduction triggers senescence (Pazmiño *et al.*, 2011). Peroxisomes have been identified as one of the main sites involved in ROS production in response to 2,4-D by the activation of xanthine oxidase and acyl-CoA oxidase (Romero-Puertas *et al.*, 2004*a*; Pazmiño *et al.*, 2011, 2014). Peroxisomes are subcellular organelles delimited by a single membrane that contain, as basic enzymatic constituents, catalase and hydrogen peroxide (H_2_O_2_)-producing flavin oxidases, and occur in almost all eukaryotic cells (Sandalio *et al.*, 2013). Peroxisomes can change their enzymatic composition, shape, size, number, and motility depending on the tissue and environmental conditions (Rodríguez-Serrano *et al.*, 2009; Sandalio *et al.*, 2013).

ROS have a double, antagonistic function in the cells depending on their concentration. That is, at low concentrations, ROS, and particularly H_2_O_2_, can act as signal molecules and regulate the expression of a large number of genes involved in cell response to different stress conditions and development (Mittler *et al.*, 2011). However, high accumulation of ROS is dangerous because it promotes oxidative damage to proteins, lipids, and nucleic acids. Oxidative damage has been demonstrated to be involved in the toxicity mechanisms of different abiotic factors (Suzuki *et al.*, 2011; Sandalio *et al.*, 2012).

In plants, nitric oxide (NO) is a key signalling molecule involved in several physiological processes from development to defence responses to both biotic and abiotic stress (Delledonne, 2005; Neill *et al.*, 2008; Astier *et al.*, 2011; del Río, 2011; Yemets *et al.*, 2011). NO can regulate diverse biological processes by directly altering proteins through oxidization, nitration, or nitrosylation (Zaninotto *et al.*, 2006; Astier *et al.* 2011; Vandelle and Delledonne, 2011). *S*-Nitrosylation refers to the binding of an NO group to a cysteine residue and can play a significant role in NO-mediated signalling (Stamler *et al.*, 2001; Astier *et al.*, 2011; Romero-Puertas *et al.*, 2013).

*In vivo* visualization of actin filaments in cells has allowed the study of numerous roles of the actin cytoskeleton in different processes in the cell. These studies have been carried out by using specific actin reporters such as the fusion protein between green fluorescent protein (GFP) and the second actin-binding domain (FABD2) of *Arabidopsis* fimbrin, AtFIM1 (GFP–FABD2; Sheahan *et al.*, 2004). The cytoskeleton governs important cell processes such as cell division and growth, vesicle transport, organelle movement, and the response of the cell to a wide range of stimuli such as light, gravity, phytohormones, pathogens, or wounding (Wasteneys and Yang, 2004; Yemets *et al.*, 2011; Lanza *et al.*, 2012; Sheremet *et al.*, 2012; Song *et al.*, 2012). The cytoskeleton has also been suggested to be one of the major targets of signalling events (Wasteneys and Yang, 2004). Recently, it has been demonstrated that the actin cytoskeleton can acts as a node of convergence in brassinosteroid (BR) and auxin signalling by regulating the bundling of actin filaments (Lanza *et al.*, 2012). A large body of evidence shows that the actin cytoskeleton plays an important role in the regulation and execution of cell expansion (Baluska *et al.*, 2001; Ketelaar *et al.*, 2004; Collings *et al.*, 2006). Dynamic actin cytoskeleton rearrangements are regulated by a pool of actin-binding proteins, which sense environmental changes and modulate the actin cytoskeleton through various biochemical activities (Hussey *et al.*, 2006; Staiger and Blanchoin, 2006; Staiger *et al.*, 2009). A number of drugs and herbicides such as dinitroanilines, benzoic acids, phosphoroamidates, pyridines, and carbamates use the cytoskeleton as a target, affecting microtubules in plant cells (Ovidi *et al.*, 2001; Blume *et al.*, 2003; Délye *et al.*, 2004). Most of these compounds alter polymerization or binding site properties of tubulin heterodimers, although the molecular mechanism is not well known (Délye *et al.*, 2004). Rahman *et al.* (2007) observed that 2,4-D and naphthylphthalamic acid removed actin and slowed down cytoplasmic streaming, although the mechanism involved was not specified. Proteomic studies have shown that plant cytoskeletal proteins can undergo many post-translational modifications including phosphorylation, *S*-glutathionylation, nitration, and *S*-nitrosylation, although their functional role and physiological relevance have yet to be elucidated (Yemets *et al.*, 2011).

For this reason, in this work, the effect of 2,4-D on the actin cytoskeleton structure and the mobility of peroxisomes and mitochondria as well as the effect of post-translational modifications of actin by oxidation and *S*-nitrosylation were analaysed. The accumulation of ROS (H_2_O_2_ and O_2_·^–^) and NO induced by 2,4-D is also studied by *in vivo* confocal imaging. It is reported that 2,4-D considerably affects the actin cytoskeleton by inducing oxidative and *S*-nitrosylated modifications on the actin, disturbing actin polymerization and compromising the dynamics of peroxisomes and mitochondria.

## Materials and methods

### Chemicals and plant materials

*Arabidopsis thaliana* (L.) ecotype Columbia was germinated after 48h incubation at 4 °C, and plants were grown in compost at 22 ºC, 16h light, and 8h darkness for 3 weeks. To study the effect of 2,4-D on *Arabidopsis* plants, the plants were sprayed once with a 23mM 2,4-D solution [prepared in 1% dimethylsulphoxide (DMSO)], and kept for 72h until analysed. Control plants were sprayed with the same concentration of DMSO used to prepare 2,4-D. The treatment time and 2,4-D concentration used in this work has been previously optimized in pea plants (Romero-Puertas *et al.*, 2004*a*). The effect of EDTA (10mM) on *Arabidopsis* leaves was studied by spraying the chemical 24h before 2,4-D treatment and the application was repeated with 2,4-D spray. To study the effect of 2,4-D on peroxisome movement, *Arabidopsis* lines expressing the fusion protein between GFP and the peroxisomal targeting signal SKL from the hydroxypyruvate reductase (GFP–SKL) were used (Rodríguez-Serrano *et al.*, 2009). The actin cytoskeleton was imaged by using the *Arabidopsis* line expressing the fusion protein GFP–FABD2 (Sheahan *et al.*, 2004). *Arabidopsis* lines simultaneously expressing cyan fluorescent protein (CFP) and yellow fluorescent protein (YFP) associated with peroxisomes and mitochondria, respectively, were obtained by cross-pollinating *Arabidopsis* marker lines px-ck and mt-yk (Nelson *et al.*, 2007) and selecting homozygous double lines. *Arabidopsis Atxdh* mutants were supplied by Dr Sagi (Ben-Gurion University, Beer Sheva, Israel) and homozygous lines were selected by analysing xanthine dehydrogenase (XDH) activity by native-PAGE and nitro blue tetrazolium staining (Pazmiño *et al.*, 2014).

### Confocal microscopy

Transgenic *Arabidopsis* leaves were sliced with razor blades and mounted between a slide and a coverslip in phosphate-buffered saline (PBS)/70% glycerol. Sections were examined using a Leica confocal laser scanning microscope, Model TCS SL (Leica Microsystems, Wetzlar, Germany). Digital images were taken across the epidermal cells. The movement of individual peroxisome stacks was analysed using the classification and particle-tracking routine of Volocity version 3.0 (Improvision; Perkin-Elmer, Palo Alto, CA, USA). This software can track the movement of individual fluorescent particles in time-resolved two or three dimensions, and automatically generates the speed and track length. For the speed analysis, the images were acquired in the *x*, *y*, *z*, and *t* dimensions. Each movie contained 15 *z*-series each containing 6–9 frames in the *z*-axis (1 μm interval; 512×512 resolution and bidirectional scan mode). The movies were generated taking 20 frames in the *x*, *y*, and *t* dimension with a 1024×1024 resolution. Quick-time movies of peroxisome movement were generated from sequential images (five frames per second). *Arabidopsis* plants expressing the fusion protein GFP–FABD2 were used to visualize the actin cytoskeleton. Images of GFP-expressing cells were acquired as a *z*-series with 1 μm interval using a Leica confocal laser scanning microscope (Exc/Em: 488/508nm) and at different times following 2,4-D (23mM) treatment (1, 24, 47, and 72h). The effect of 25 μM latrunculin B (Lat B, an inhibitor of actin polymerization, prepared in 0.2% DMSO) on the actin cytoskeleton was also studied in GFP–FABD2 *Arabidopsis* plants treated with these compounds for 45min.

### Analysis of H_2_O_2_ and NO in plants extracts

The H_2_O_2_ concentration was determined in acid extracts from *Arabidopsis* leaves by spectrofluorimetry as described by Pazmiño *et al*. (2011). All processes were conducted at 4 ºC. Leaves (0.5g) were extracted with 1.5ml of 1M HClO_4_, in the presence of insoluble PVP (polyvinylpyrrolidone; 5%) and centrifuged at 12 000 *g* for 10min (4 ºC); the supernatant was filtered through a 0.45 μm Millipore filter. The pH was adjusted to 7.0 with 5M K_2_CO_3_ and the filtrate was finally centrifuged at 12 000 *g* for 2min to remove KClO_4_. The supernatant was used to measure the H_2_O_2_ by spectrofluorimetry using homovanillinic acid (Ex/Em: 325/425nm) and horseradish peroxidase (HRP).

NO was analysed by fluorimetry using 4,5-diaminofluorescein (DAF-2), as described by Nakatsubo *et al.* (1998). After treatment with 2,4-D, leaf extracts were made and incubated with DAF-2 in 50mM HEPES buffer, pH 7.5, for 2h at 37 ºC. Afterwards, NO was measured by analysing DAF-2 fluorescence (Ex/Em: 495/515nm).

### ROS and NO detection by confocal laser scanning fluorescence microscopy

ROS and NO accumulation were imaged by confocal laser scanning microscopy (CLSM). Superoxide radicals were detected by incubating leaf sections with 10 μM dihydroethidium (DHE; Fluka, Buchs, Switzerland; Ex/Em: 450–490/520nm) in 10mM TRIS-HCl (pH 7.4), for 30min at 37 ºC, as describeed by Sandalio *et al.* (2008). H_2_O_2_ was detected by using 2′,7′-dichlorodihydrofluorescein diacetate (DCF-DA) in 10mM TRIS-HCl (pH 7.4) for 30min at 37 ºC, and NO was detected with 4,5-diaminofluorescein diacetate (DAF-2DA) for 1h at 25 ºC as indicated by Sandalio *et al.* (2008). As a negative control, 2-(4-carboxyphenyl)-4,4,5,5-tetramethylimidazoline-1-oxyl-3-oxide (cPTIO; 2mM) was used as an NO scavenger. After leaves were embedded in 30% (w/v) polyacrylamide blocks, sections were cut by a vibratome and mounted for examination with a confocal laser scanning microscope (Leica TCS SL; Leica Microsystems). Fluorescence was quantified using LAS AF Leica software and expressed as arbitrary units.

### Histochemical analyses

For histochemical analyses of H_2_O_2_, leaves from control and 2,4-D-treated plants were excised and immersed in a 1% solution of 3,3′-diaminobenzidine (DAB) in 10mM MES buffer (pH 6.5), vacuum-infiltrated for 5min, and then incubated at room temperature for 8h in the absence of light. Leaves were illuminated until the appearance of brown spots characteristic of the reaction of DAB with H_2_O_2_. Leaves were bleached by immersion in boiling ethanol to visualize the brown spots (Romero-Puertas *et al.*, 2004*b*). Cell death was evaluated by histochemical analysis using Trypan Blue (Koch and Slusarenko, 1990) at different times during treatment.

### Western blot analysis

To analyse the effect of 2,4-D on GFP–fimbrin and actin expression, leaves were homogenized in buffer containing 50mM TRIS-HCl (pH 7.8), 0.1mM EDTA (0.2% v/v) Triton X-100, and protease inhibitor cocktail (Sigma, St. Louis, MO, USA). Homogenates were centrifuged at 16 000 *g* for 30min at 4 ºC. Equal amounts of proteins were loaded for SDS–PAGE (12% acrylamide) and transferred onto a polyvinylidene fluoride (PVDF) membrane (Millipore Co., Bedford, MA, USA) in a Bio-Rad Semi-Dry Transfer Cell (Bio-Rad, Hercules, CA, USA). GFP was detected using anti-GFP monoclonal antibody (Clontech; 1/10 000 dilution) and goat anti-mouse IgG conjugated with HRP as secondary antibody (Bio-Rad; 1/ 10 000 dilution). Actin was detected using a specific polyclonal antibody (1/1000 dilution, Molecular Probes™) and IgG anti-rabbit–HRP (Bio-Rad; 1/10 000 dilution). To analyse the total amount of filamentous actin (F-actin) versus free globular actin (G-actin), leaves were homogenized in buffer containing 0.1M PIPES (pH 6.9) 30% (v/v) glycerol, 5% (w/v) DMSO, 1mM MgSO_4_, 1mM EGTA, 1% (v/v) Triton X-100, 1mM ATP, and protease inhibitor cocktail. Homogenates were centrifuged at 16 000 *g* for 75min at 4 ºC to separate F-actin from G-actin. F-actin from the pellet was depolymerized with cytochalasin and solubilized in an equal volume of supernatant containing 0.1M PIPES (pH 6.9), 1mM MgSO_4_, 10mM CaCl_2_, and 5 μM cytochalasin D. After incubation for 1h, equal volumes of both fractions were analysed by western blot using a specific antibody against actin as mentioned above (Rasmussen *et al.*, 2010).

### Immunochemical detection of *S*-nitrosylated actin

*S*-Nitrosylated proteins were detected following the biotin-switch method that converts –SNO into biotinylated groups (Jaffrey *et al.*, 2001). *Arabidopsis* leaves were homogenized in MAE buffer (25mM HEPES, 1mM EDTA, 0.1mM neocuproine, 0.2% Triton X-100, pH 7.7) containing complete protease inhibitor cocktail (Sigma). The extract was centrifuged at 4 °C for 30min. Proteins were then assayed with the biotin-switch method. Briefly, an equal amount of protein (300 μg) from control and treated plant leaf extracts was subjected to the biotin-switch assay (Ortega-Galisteo *et al.*, 2012) and biotinylated proteins were purified by immunoprecipitation overnight at 4 ºC with 15 μl of IPA (UltralLink Immobilized Protein A/G Pierce) mg^–1^ of protein and pre-incubated with 2 μl of anti-biotin antibody (Sigma). Beads were washed three times with PBS, and bound proteins were eluted with 10mM dithiothreitol (DTT) in SDS–PAGE solubilization buffer, loaded on a 12% SDS–polyacrylamide gel, transferred to a PVDF (polyvinylidene fluoride) membrane, and actin was detected with specific antibodies (1/1000 dilution, Molecular Probes™).

### Immunochemical detection of oxidative modified actin

The proteins containing carbonyl groups were identified as described by Romero-Puertas *et al.* (2002). Equal amount of proteins (500 μg) from leaf extracts were derivatized with 10mM 2,4-dinitrophenylhydrazine (DNPH; Sigma-Aldrich Co.) and immunoprecipitated with antibodies against DNP linked to IPA overnight at 4 ºC. Oxidized-purified proteins (10 μl) were subjected to SDS–PAGE (12% acrylamide) and transferred onto PVDF membranes as mentioned above. Actin was detected using specific antibodies (1/1000 dilution, Molecular Probes™).

### Protein and statistical analysis

Protein concentration was determined with the Bio-Rad Bradford Protein Assay kit using bovine serum albumin (BSA) as standard. Data were subjected to one-way analysis of variance for each parameter. When the effect was significant (*P*>0.05), differences among means were evaluated for significance by Duncan’s multiple-range test (*P*>0.05).

## Results

### Effect of 2,4-D on *Arabidopsis* leaf phenotype and oxygen and nitrogen reactive species accumulation

**Fig. 1.**
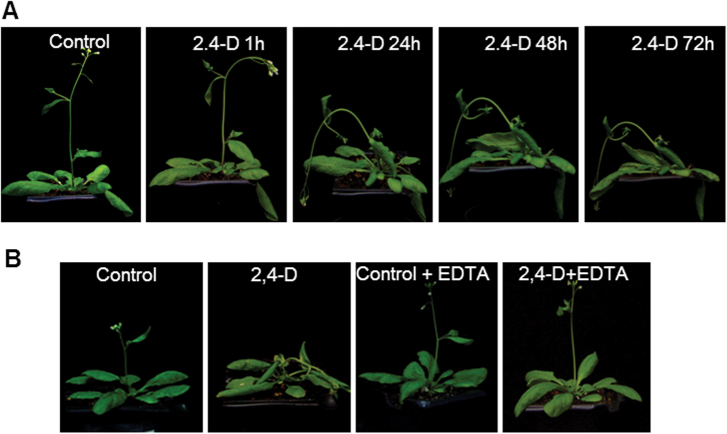
Effect of 2,4-D and EDTA on *Arabidopsis* phenotype. (A) Plants were treated once by foliar application of 23mM 2,4-D and the effect on phenotype was followed after different periods of treatment (1–72h). Images correspond to the same plant at different treatment times. (B) Effect of EDTA (10mM) on 2,4-D-induced phenotype. Plants were sprayed with EDTA before treatment with 2,4-D and the effect was analysed after 72h of treatment.

In previous work, the concentration of the herbicide 2,4-D and the time of treatment was optimized in order to visualize its toxic effects on pea plants, with 23mM 2,4-D and 72h of treatment being the experimental conditions selected (Romero-Puertas *et al.*, 2004*a*). Therefore, these conditions were used to carry out the experiments in *Arabidopsis* plants. The supply of 23mM 2,4-D to *Arabidopsis* plants produced a severe curling or epinasty of rosette leaves, loss of leaf turgidity, and curling of the flower stem which started after 1h, reaching a maximum after 72h of treatment (Fig. 1A; Supplementary Fig. S1A available at *JXB* online). This effect was reduced by the treatment with EDTA (Fig. 1B), as was shown in previous work on pea leaves (Pazmiño, 2009, 2014).

**Fig. 2.**
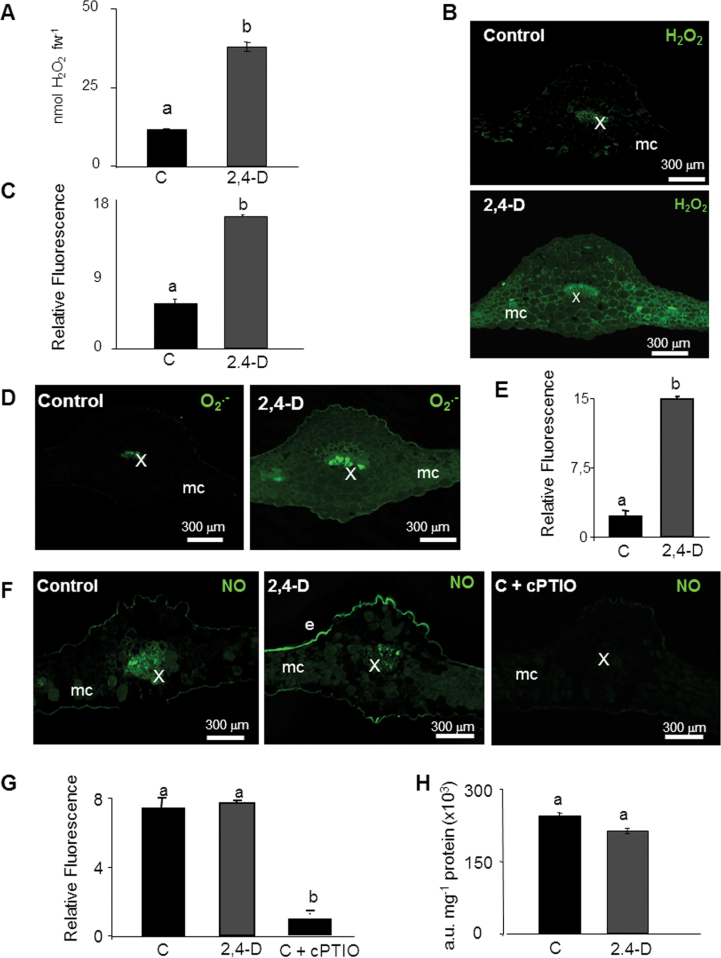
Imaging and quantification of H_2_O_2_, O_2_·^–^, and NO production induced by the treatment with 2,4-D in *Arabidopsis thaliana* leaves. (A) H_2_O_2_ content was analysed in acid extracts from *Arabidopsis* leaves by fluorimetry. Values are means ±SE of four different experiments with three independent extracts each. (B) Imaging of H_2_O_2_ accumulation in cross-sections of *Arabidopsis* leaves by CLSM using DCF-DA (Ex/Em: 485/530nm). DCF-DA fluorescence was quantified in arbitrary units (C). (D, E) Imaging and quantification of O_2_·^–^ production using DHE (Ex/Em: 450–490/520nm. (F, G) Imaging and quantification of NO production using DAF-2DA (Ex/Em: 495/515nm). cPTIO was used as an NO scavenger. (H) NO content was quantified in arbitrary units (a.u.) in leaf extracts from *Arabidopsis* leaves by fluorimetry. Images are maximal projections from several optical sections and are representative of at least 15 leaf sections from four different experiments. Different letters indicate a significant difference at *P*<0.05 as determined by Duncan′s multiple-range test. e, epidermis; mc, mesophyll cells; st, stomata; x, xylem.

The analysis of total H_2_O_2_ in *Arabidopsis* leaf extracts after treatment with 2,4-D shows a 2-fold increase in H_2_O_2_ (Fig. 2A). By using histochemistry with DAB, a strong increase of H_2_O_2_ was detected in 2,4-D-treated plants in comparison with untreated plants, the highest accumulation being registered in vascular tissues (Supplementary Fig. S1B at *JXB* online). The accumulation of H_2_O_2_ was also studied in leaf cross-sections using DCF-DA and CLSM. 2,4-D induced an increase in DCF fluorescence, associated mainly with mesophyll cells, although an increase in fluorescence in secondary veins also appeared in 2,4-D-treated leaves (Fig. 2B, C). The analysis of O_2_·^–^ in cross-sections of *Arabidopsis* leaves showed an induction of O_2_·^–^ by the herbicide (Fig. 2D, E) which was reversed by incubation with superoxide dismutase (SOD; data not shown). The O_2_·^–^ accumulated in the main and secondary veins, but also in mesophyll and epidermal cells (Fig. 2D; Supplementary Fig. S2). A higher magnification of mesophyll cells revealed the O_2_·^–^-dependent fluorescence associated mainly in small puncta which could represent localization to small organelles such as mitochondria and peroxisomes, while neither chloroplasts nor plasma membrane show any DHE signal (Supplementary Fig. S2). In turn, the image of NO accumulation displayed by DAF-2D fluorescence showed no apparent differences due to the treatment with 2,4-D in terms of total NO accumulation, although a slight increase in fluorescence was observed in the epidermis (Fig. 2F, G). The NO scavenger cPTIO was used as a negative control, showing a considerable reduction of DAF-2DA fluorescence (Fig. 2F, G). NO production was analysed using spectrofluorimetry in order to quantify changes in NO accumulation by the herbicide, but no changes were found in comparison with the values in untreated plants (Fig. 2H).

### 2,4-D disturbs the actin cytoskeleton by post-translational changes in actin

**Fig. 3.**
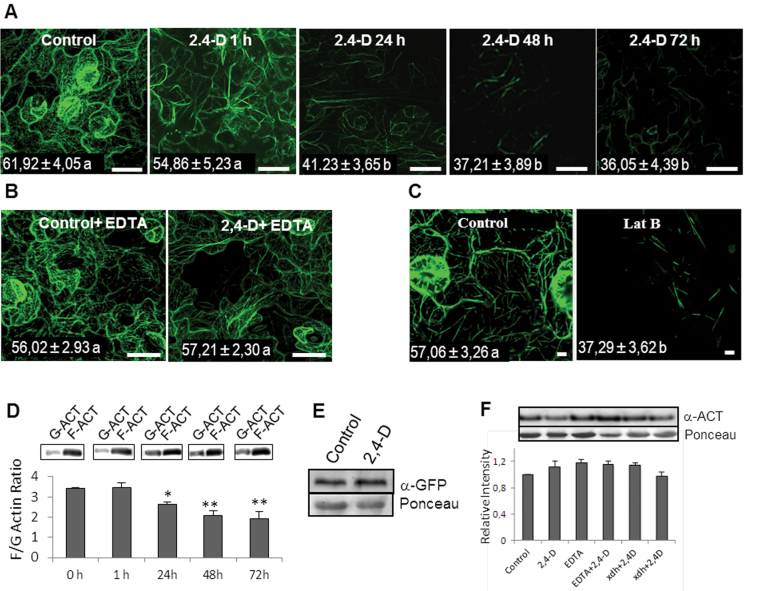
Effect of 2,4-D on the actin cytoskeleton in epidermal cells of *Arabidopsis* leaves. (A) *Arabidopsis* plants expressing the fusion protein GFP–FABD2 were used to visualize the effect of 2,4-D (23mM) on the actin cytoskeleton at different treatment times. (B) Effect of EDTA (10mM) and 2,4-D on the actin cytoskeleton after 72h of treatment. (C) Effect of latrunculin B (Lat B; 25 μM) on the actin cytoskeleton. Fluorescence for each treatment was quantified as indicated in the Materials and methods and expressed in arbitrary units. The mean ±SE of at least 10 leaf sections from three different experiments is shown inside the panels. Data followed by the same latter are not statistically different according to Duncan’s multiple-range test. Bars represent 25 μm in A and B, and 5 μm in C. (D) Effect of 2,4-D on the F-actin/G-actin ratio during the treatment, analysed by western blot of proteins using anti-actin antibodies. An equal volume of proteins was used for each fraction. (E) GFP–FABD2 expression in leaves after 72h of treatment analysed by western blot of proteins using a specific anti-GFP antibody. An equal amount of protein was loaded per well. (F) Variation in total actin protein accumulation in WT plants after 72h of treatment with 2,4-D and EDTA and in *Atxdh* plants monitored by western blot analysis using a specific anti-actin antibody. An equal amount of protein was loaded per well. (This figure is available in colour at *JXB* online.)

Recently Rahman *et al.* (2007) have shown that 2,4-D can affect actin cytoskeleton structure, although the mechanism involved has not been established so far. To look at this in more depth, in this study the effect of this chemical on the structure of the actin cytoskeleton over time was analysed by using a transgenic *Arabidopsis* line expressing GFP associated with an actin-binding protein (GFP–FABD2; Sheahan *et al.*, 2004). The analysis revealed a slight but not significant reduction of GFP associated with actin filaments after 1h of treatment, and after 24h a significant reduction was observed. The maximum effect was observed after 72h, which revealed a reduction in the number and thickness of actin filaments (Fig. 3A). EDTA prevented the disturbances to the organization of the actin cytoskeleton (Fig. 3B) in the same way as it prevented epinasty. To characterize the disturbances of the actin cytoskeleton induced by 2,4-D, GFP–FABD2 plants were treated with Lat B, which is a well known inhibitor of actin polymerization (Sheahan *et al.*, 2004). Lat B produced a severe reduction in most of the filamentous actin after 45min of treatment, showing a similar image to that observed with 2,4-D, which suggests that the changes observed in the actin filament network induced by this herbicide could be due to a reduction in the ability of actin to polymerize (Fig. 3C). This fact was studied by analysing the content of G-actin and F-actin in leaf extracts at different treatment times. A statistically significant reduction in the F-actin/G-actin ratio was observed after 24h of treatment, with a maximum after 72h of treatment (Fig. 3D). To study if the disturbance in the actin cytoskeleton is associated with cell death, leaves of *Arabidopsis* plants were stained with Trypan Blue, a marker of cell viability, at different treatment times (24, 48, and 72h). The results obtained did not show any cell death due to the treatment with 2,4-D even after 72h (Supplementary Fig. S3 at *JXB* online). In addition to this, to rule out degradation processes affecting GFP–FABD2 during the treatment with 2,4-D, a western blot analysis was carried out using a monoclonal anti-GFP antibody. The results obtained shown that the content of GFP–FABD2 was not affected by the treatment with 2,4-D for 72h (Fig. 3E). The total actin present in extracts was also analysed by western blot using specific antibodies against actin. No differences were detected in terms of total protein between control and 2,4-D-treated plants after 72h of treatment, demonstrating that actin is not down-regulated or proteolitically degraded by the 2,4-D treatment (Fig. 3F). The same results were obtained in plants treated with EDTA.

**Fig. 4.**
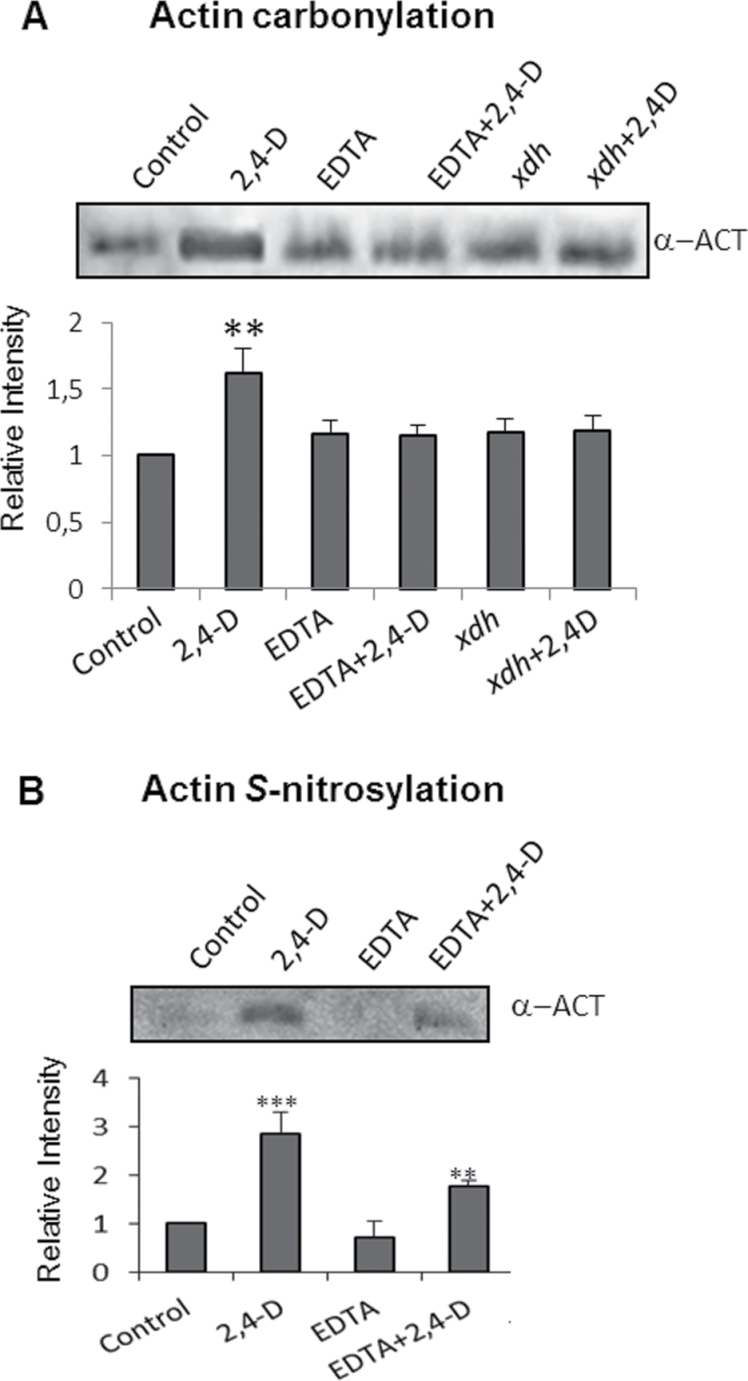
Analysis of post-translational modifications of actin by carbonylation and *S*-nitrosylation. (A) Detection of carbonylated actin. Proteins from leaf extracts (500 μg) were derivatized with DNPH and immunoprecipitated with anti-DNP-IPA as indicated in the Materials and methods. Oxidized-purified proteins (10 μl) were subjected to SDS–PAGE, transferred onto PVDF membranes, and analysed with an anti-actin antibody. The figure is representative of four independent experiments. (B) Detection of *S*-nitrosylated actin. *S*-Nitrosylated proteins were labelled with biotin and immunopurified with anti-biotin–IPA, separated by SDS–PAGE, and the actin was identify by western blot analysis using an anti-actin antibody. The figure is representative of three independent experiments.

In previous work, it was demonstrated that xanthine oxidoreductase (XOD/XDH) is involved in ROS production induced by 2,4-D (Pazmiño *et al.*, 2014), and *Arabidopsis* mutants deficient in this protein (*Atxdh*) show a significant reduction of epinasty induced by 2,4-D; for this reason the effect of 2,4-D on the content of actin in this mutant was analysed. No differences were observed between the wild type (WT) and *Atxdh* (Fig. 3F). To study the cause of 2,4-D-dependent disturbances in the structure of the actin cytoskeleton, post-translational modifications of actin by oxidation and *S*-nitrosylation were analysed after 72h of 2,4-D exposure. Leaf extracts from WT and *Atxdh* plants treated with 2,4-D for 72h were incubated with 10mM DNPH, and oxidized proteins containing carbonyl groups were purified by immunoprecipitation using antibodies against DNP linked to IPA; actin was visualized by western blot using a specific antibody. The results obtained revealed a strong increase in oxidized actin in 2,4-D-treated plants which was considerably reduced in *Atxdh* lines and the WT treated with EDTA (Fig. 4A). These results indicate that ROS production stimulated by 2,4-D is involved in oxidative alterations of actin, which in turn would promote disturbances in the actin cytoskeleton ultrastructure. Actin reportedly undergoes *S-*nitrosylation in both animal and plant tissue, and in animal tissue this change has been demonstrated to affect the rate of actin polymerization under oxidative stress (Dalle-Donne *et al.*, 2000). Therefore, *S*-nitrosylated proteins from *Arabidopsis* leaf extracts were analysed by the biotin-switch method and purified by immunoprecipitation using anti-biotin antibody–IPA, and actin was identified with specific antibodies. The treatment with 2,4-D boosted the content of *S*-nitrosylated actin in comparison with untreated control plants, and EDTA significantly reduced it (Fig. 4B).

### Peroxisomal dynamics is affected by 2,4-D

**Fig. 5.**
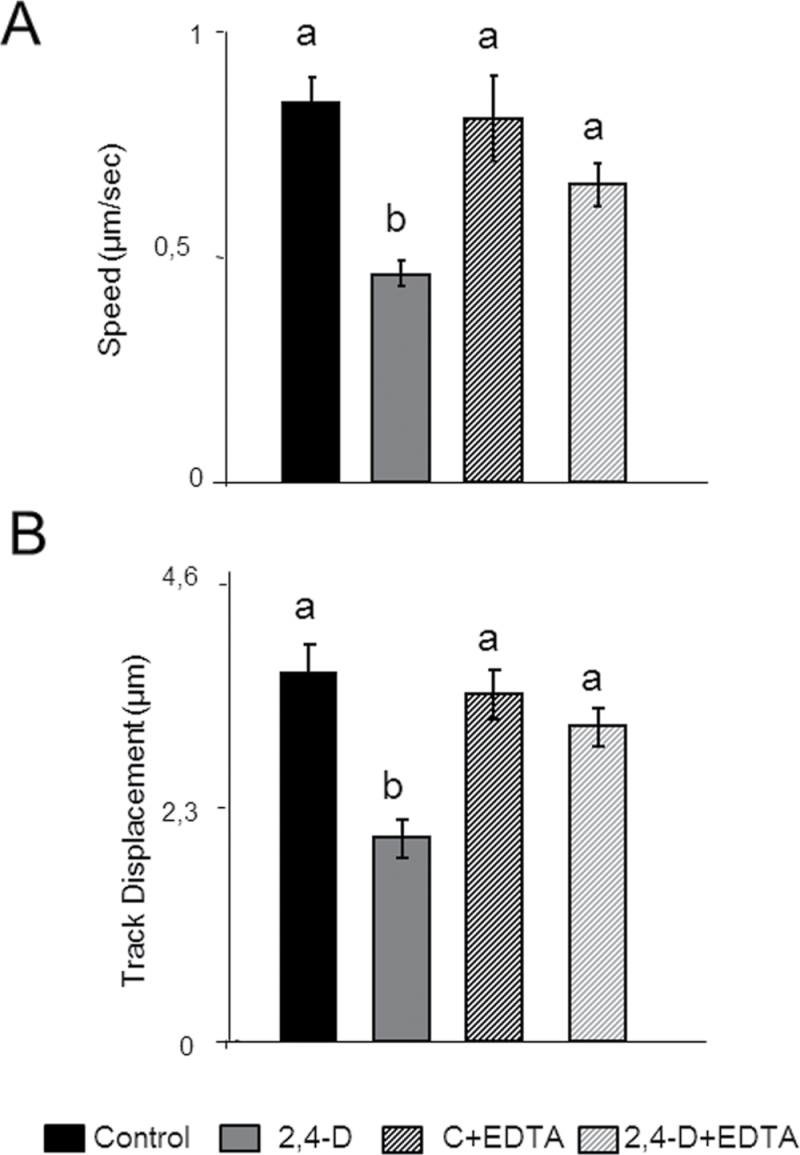
Effect of 2,4-D on peroxisome dynamics in epidermal cells. Seedlings expressing GFP–SKL were treated with 23mM 2,4-D with or without 10mM EDTA for 72h. The speed (A) and displacement (B) were studied by time-lapse analysis using a confocal laser microscope, and the images obtained were processed using Volocity3 software. Results are means ±SE of three different parts of the leaf, and at least 10 different plants from three different experiments were used. Values with different letters are significantly different (*P*<0.05) as determined by Duncan’s multiple-range test.

Because peroxisomes move along the actin cytoskeleton (Mano *et al.*, 2002; Van Gestel *et al.*, 2002) the dynamics of these organelle under 2,4-D toxicity was studied. Peroxisome movement in epidermal cells was assessed after 72h of treatment. The herbicide caused a 2-fold reduction of speed (Fig. 5A) and a reduction of the displacement rate of these organelles (Fig. 5B). Movies of control and 2,4-D-treated plants showing differences in the dynamics of peroxisomes are provided in Video S1A, B and S2A, B at *JXB* online. As mentioned previously, EDTA can reduced the disturbances of the actin cytoskeleton induced by 2,4-D, and therefore the role of EDTA in peroxisomal movement was also investigated. The treatment with EDTA reversed the effect of 2,4-D on both the speed and displacement of peroxisomes, reaching values similar to those of the untreated leaves (Fig. 5A, B). In order to determine if the effect of 2,4-D is specific for peroxisomes, or is a general effect on organelle motility, the effect of 2,4-D on the movement of mitochondria in *Arabidopsis* lines expressing YFP in mitochondria and CFP associated with peroxisomes was analysed. The dynamics of mitochondria was also disturbed by the herbicide, showing a severe reduction in their motility (Supplementary Video S2A, B).

## Discussion

Auxins regulate a number of processes related to development and growth in plants, being involved in cell elongation, tissue differentiation, tissue polarity, or leaf expansion (Benjamins and Scheres, 2008; Delker *et al.*, 2008). 2,4-D is a synthetic auxin specific for dicotyledons and is considered to be among the most successful herbicides used in agriculture (Grossmann, 2010; Pazmiño *et al.*, 2012). One of the most characteristic effects of 2,4-D on sensitive plants is the development of epinasty and stem curvature, as well as reduction of root and stem growth (Grossmann, 2010; Pazmiño *et al.*, 2011, 2012). Different studies have demonstrated that the toxicity of this herbicide is mediated by uncoupling oxidative phosphorylation, changes in the plasma membrane potential, or oxidative stress (Grossmann *et al.*, 2001; Pazmiño *et al.*, 2012). Recently, it was demonstrated that ROS are involved in the toxicity of 2,4-D, being responsible for both the epinasty and senescence induced by this herbicide (Pazmiño *et al.*, 2011, 2014). The analysis of different sources of ROS under 2,4-D toxicity point to XOD/XDH and acyl-CoA oxidase (ACX) as the main agents responsible for the oxidative stress imposed by 2,4-D (Pazmiño *et al.*, 2011, 2014). Both enzymes are components of peroxisomes, which are characterized by a strong oxidative metabolism (Sandalio *et al.*, 2013). The role of peroxisomes in ROS and NO metabolism has recently been demonstrated, and the importance of these molecules in signalling has been elucidated over the past 10 years (Vandenabeele *et al.*, 2004; del Río 2011; Ortega-Galisteo *et al.*, 2012; Sandalio *et al.*, 2013). The rate of ROS accumulation would define the role of these reactive species as signal molecules (low production) or as dangerous compounds (high accumulation) (Mittler *et al.*, 2011; Sandalio *et al.*, 2013). In turn, NO can interfere with signal transduction pathways or can modify proteins, modulating their activities or properties (Moreau *et al.*, 2010). The analysis of H_2_O_2_ accumulation by different methods showed that 2,4-D induced the overaccumulation of this ROS, which in turn caused oxidative alteration of proteins, as was demonstrated recently by carbonyl content analyses (Pazmiño *et al.*, 2014). One of the target proteins of this oxidative modification was actin. Concerning the main sources of ROS under these conditions, the analyses of ROS accumulation by CLSM suggest that peroxisomes and mitochondria may be the main cell compartments involved in ROS production. Recently, the production of H_2_O_2_ in peroxisomes induced by 2,4-D in tobacco leaves transiently expressing the biosensor HyperAs-SKL in peroxisomes and pea leaf peroxisomes was reported (Sandalio *et al.*, 2013; Pazmiño *et al.*, 2014). The generation of ROS and particularly ·OH is a prerequisite for cell wall loosening and normal growth (Schopfer *et al.*, 2002; Liszkay *et al.*, 2004) and, under the conditions used in this work, 2,4-D could promote overaccumulation of these species, which in turn could trigger cell malformation, leading to epinasty, a hypothesis supported by the protective role of EDTA which can act as a metal chelator, avoiding Fenton-type reactions, and also reacts directly with ·OH at a rate constant of 2.8×10^–9^ (Halliwell and Gutteridge, 2007).

The plants treated with 2,4-D showed a strong reduction in actin bundling and polymerization, which increase with the time of treatment and were completely prevented by EDTA. EDTA also prevented epinasty, demonstrating that the cytoskeletal disturbances are involved in the development of this phenotype. The protective effect of EDTA was due mainly to the reduction of actin oxidation. Recently it was observed that EDTA reduces oxidation of proteins in *Arabidopsis* plants treated with 2,4-D (Pazmiño *et al.*, 2014) and also reduced the accumulation of H_2_O_2_ induced by 2,4-D in pea shoots (Pazmiño *et al.*, 2014). These results suggest that ·OH is the main ROS involved in the actin cytoskeleton disturbances, and the results obtained with *Atxdh* plants suggest that XOD/XDH are partially involved in the production of this ROS. In addition to ROS, NO is also a key molecule involved in signalling and controlling functionality of different proteins by combining with cysteines and giving rise to *S*-nitrosylation of proteins. Despite the absence of changes in total NO accumulation, an increase of *S*-nitrosylation of actin was observed in 2,4-D-treated plants when *S*-nitrosylated proteins were purified. A reduction of *S*-nitrosylation was observed when plants were pre-treated with EDTA however. It appears that this decrease it is not metal dependent as EDTA would favour and protect *S*-nitrosylation (Jaffrey *et al.*, 2001). Other molecules, however, could also regulate this post-translational modification, such as ascorbate or glutathione, whose concentration is altered by EDTA and 2,4-D treatment in pea plants (Pazmiño, 2009). Studies carried out *in vitro* and in animal cells have shown that actin is a major target of different post-translational modifications, such as oxidation and *S*-nitrosylation, and both processes triggered disturbances in actin polymerization, giving rise to changes in cell morphology through the formation of multiple surface blebbs on the plasma membrane (Dalle-Donne *et al.*, 2001). Similar changes in cell morphology have also been observed in pea leaves treated with 2,4-D (Pazmiño *et al.*, 2011). Actin has several cysteines susceptible to redox modifications and also has several methionines susceptible to oxidation (Terman and Kashina, 2013). However, the role and hierarchical relationship between these post-translational modifications of actin under physiological and stress conditions have not being established so far (Terman and Kashina, 2013). The interplay between post-translational modifications of proteins has emerged as a very important regulatory mechanism (Sun *et al.*, 2006; Lounifi *et al.*, 2013). ROS and NO can compete for the same cysteine residues to regulate proteins, and therefore can exert antagonistic roles. *S*-Nitrosylation has been suggested to protect proteins against irreversible carbonylation, and, in its turn, irreversible oxidation of thiols can block the physiological modification by *S*-nitrosylation (Sun *et al.*, 2006; Lounifi *et al.*, 2013). In addition to post-translational modification of actin in animal cells, it has been reported that ROS can also disturb the actin cytoskeleton by activating mitogen-activated protein kinases, which lead to the phosphorylation of F-actin, affecting actin polymerization and cytoskeleton dynamics (Dalle-Donne *et al.*, 2001; Foissner *et al.*, 2002).

There is a cross-talk between auxins and actin, and a self-referring regulatory circuit between polar auxin transport and actin organization has been reported, although the mechanism is not well understood (Dhonukshe *et al.*, 2008; Nick *et al.*, 2009). Auxin transport inhibitors, such as 2,3,5-triiodobenzoic acid (TIBA) or sodium 4-phenylbutyrate (PBA), repress vesicle trafficking by influencing the actin cytoskeleton (Dhonukshe *et al.*, 2008), and exogenous IAA regulates actin bundling, promoting the transformation of massive longitudinal bundles into finer strands (Nick *et al.*, 2009), although the mechanism involved in these changes of the actin cytoskeleton have not been demonstrated. More recently, it was observed that BRs induced a wavy phenotype in *Arabidopsis* roots which was due to changes in the distribution of actin filaments and their dynamics (Lanza *et al.*, 2012). NO can induce actin depolymerization in sycamore tree cells treated with fusicoccin, and this process has also been associated with the induction of programmed cell death (Malerba *et al.*, 2008). ROS and NO also mediated actin reorganization and the induction of programmed cell death in the pollen self-incompatibility response of *Papaver*, although the molecular mechanisms have not established so far (Wilkins *et al.*, 2011). In the present work, it has been demonstrated that 2,4-D does not induce degradation or down-regulation of actin and GFP–FABD2 but induces actin modifications by oxidation and *S-*nitrosylation, which affect the polymerization of F-actin, leading to a strong reduction of actin organization. In animal cells, *S*-nitrosylation interferes with the normal state of F-actin, resulting in depolymerization (Dalle-Donne *et al.*, 2000). *S*-Nitrosylated G-actin polymerizes less efficiently than control actin and forms a lower amount of F-actin, compared with unmodified actin, which shortens the actin length distribution (Dalle-Donne *et al.*, 2000). In maize roots, exogenous NO donors reportedly disturbed the actin cytoskeleton and vesicle trafficking by reorganization of F-actin, and this effect was specific for cell type and developmental state (Kasprowicz *et al.*, 2009).

The disturbances observed in the actin cytoskeleton could be responsible for the leaf epinasty induced by 2,4-D, and, in fact, actin has been demonstrated to regulate the growth and the shape of leaf epidermal pavement cells and trichomes (Smith and Oppenheimer, 2005), and has been found to be involved in growth alterations (Ketelaar *et al.*, 2004). Baluska *et al.* (2001) have also observed that a reduction of the actin cytoskeleton induces cell radial growth, resulting in the cells being shorter and wider, which could explain the epinasty induced by 2,4-D observed in this work. In addition to this, the disturbances in the actin cytoskeleton promote a reduction of the mobility and displacement of peroxisomes in response to 2,4-D. This effect was not specific for peroxisomes, and the motility of mitochondria was also affected by 2,4-D. These disturbances could considerably affect the metabolism of these organelles because they share several metabolites with each other and with chloroplasts, and the disruption of their dynamics could compromise the metabolic pathways where they are involved. Thus, 2,4-D promotes reduction of carbon fixation and starch formation in plants (Grossmann, 2010), and affects mitochondrial respiration and fatty acid β-oxidation in yeast, animal, and plant cells (Teixeira *et al.*, 2007; Romero-Puertas *et al.*, 2004*a*; Grossmann, 2010). Peroxisomes contain a large battery of antioxidants and can participate in removing ROS from different parts of the cells, and disturbances in their mobility could limit their role in antioxidative defence.

**Fig. 6.**
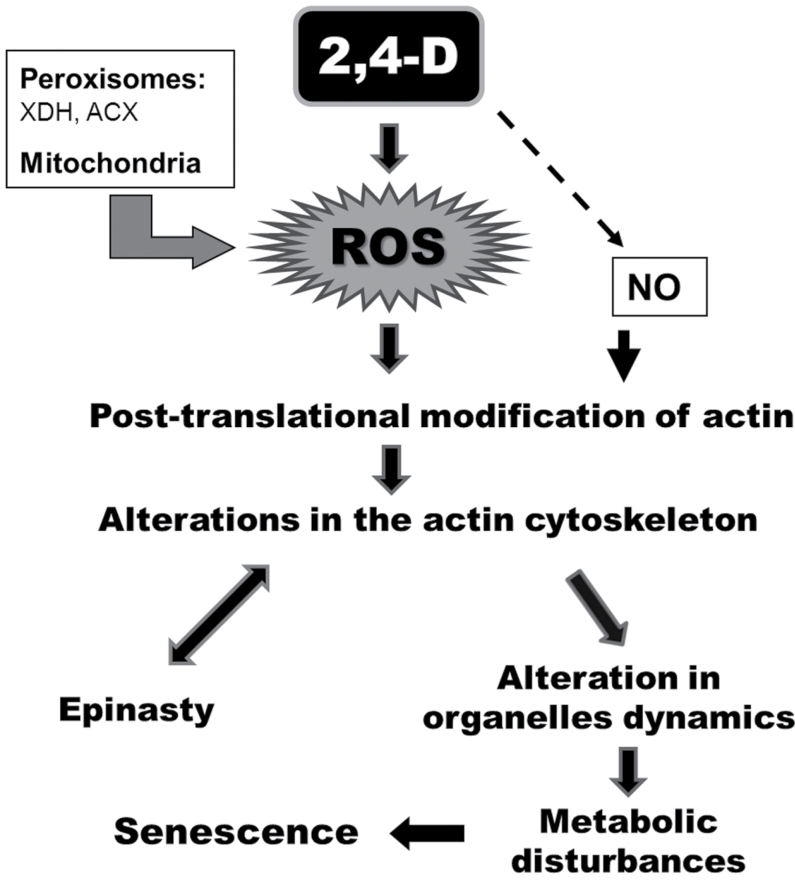
Schematic showing the possible mechanistic toxicity of 2,4-D in *Arabidopsis* plants. 2,4-D promotes oxidative stress, where peroxisomes and mitochondria represent the main sources of ROS, giving rise to post-translational changes of actin by oxidation and *S*-nitrosylation, causing disturbances in the actin cytoskeleton and thereby affecting trafficking of organelles. These structural changes in turn appear to be responsible for leaf epinasty, although processes involved in developing epinasty can also contribute to actin cytoskeleton disturbances. Alteration of the cytoskeleton could also be responsible for metabolic disturbances, signalling disruption, and further senescence. XDH, xanthine dehydrogenase; ACX, acyl-CoA oxidase.

In conclusion, 2,4-D promotes oxidative stress, giving rise to post-translational changes of actin by oxidation and *S*-nitrosylation, causing disturbances in the actin cytoskeleton and thereby affecting the dynamics and metabolism of peroxisomes and mitochondria. These structural changes in turn appear to be responsible for epinastic deformation of the leaf characteristic of this herbicide. Disturbances in the actin cytoskeleton could also affect vesicle trafficking and, in general, organelle movement, giving rise to the metabolic disturbances and even further cell death after very long exposure to the herbicide (Fig. 6).

## Supplementary data

Supplementary data are available at *JXB* online.

Figure S1. 2,4-D produces epinasty in *Arabidopsis* leaves and accumulation of H_2_O_2_ mainly in vascular tissue.

Figure S2. Imaging of O_2_·^–^ production by CLSM using DHE (Ex/Em: 450–490/520nm, green) and chlorophyll autofluorescence (red) showing magnifications of mesophyll cells from 2,4-D-treated plants.

Figure S3. Histochemical staining with Trypan Blue of *Arabiodopsis* leaves treated for different times with 2,4-D (23mM).

Video S1A. Movies showing peroxisomal dynamics in epidermal cells from control *Arabidopsis* plants expressing GFP–SKL.

Video S1B. Movies showing the effect of 23mM 2,4-D on peroxisomal dynamics in epidermal cells from control *Arabidopsis* plants expressing GFP–SKL.

Video S2A. Movies showing peroxisomal and mitochondrial dynamics in epidermal cells from control double marker *Arabidopsis* px-ck×mt-yk plants.

Video S2B. Movies showing the effect of 2,4-D on peroxisomal and mitochondrial dynamics in epidermal cells from double marker *Arabidopsis* px-ck×mt-yk plants.

Supplementary Data

## Abbreviations:

ACX: acyl-CoA oxidase
BR: brassinosteroid
CFP: cyan fluorescent protein
2,4-D: 2,4-dichlorophenoxyacetic acid
cPTIO: 2-(4-carboxyphenyl)-4,4,5,5-tetramethylimidazoline-1-oxyl-3-oxide
DAB: 3,3′-diaminobenzidine
DAF-2: 4,5-diaminofluorescein
DCF-DA: 2′,7′-dichlorodihydrofluorescein diacetate
DHE: dihydroethidium
DMSO: dimethylsulphoxide
DNPH: 2,4-dinitrophenylhydrazine
FABD2: actin-binding domain 2 of fimbrin
GFP: green fluorescent protein
HRP: horseradish peroxidase
IAA: indole-3-acetic acid (natural auxin)
IPA: immobilized protein A
Lat B: latrunculin B
ROS: reactive oxygen species
SOD: superoxide dismutase
TIBA: 2,3,5-triiodobenzoic acid
XDH: xanthine dehydrogenase
XOD: xanthine oxidase
YFP: yellow fluorescent protein.
