# Supplementary material for: 2,4-Dichlorophenoxyacetic acid promotes S-nitrosylation and oxidation of actin affecting cytoskeleton and peroxisomal dynamics
Source: J Exp Bot. 2014 Jun 9;65(17):4783–93. doi: 10.1093/jxb/eru237 (PMC4144765; doi:10.1093/jxb/eru237)
Supplement: Supplementary Data [file supp_65_17_4783__index.html]

2,4-Dichlorophenoxyacetic acid promotes S-nitrosylation and oxidation of actin affecting cytoskeleton and peroxisomal dynamics — Supplementary Data 

# 2,4-Dichlorophenoxyacetic acid promotes *S*-nitrosylation and oxidation of actin affecting cytoskeleton and peroxisomal dynamics

## Supplementary Data

Data files

**Files in this Data Supplement:**

- Supplementary Data - Supplementary Data
- Supplementary Data - Supplementary Data
- Supplementary Data - Supplementary Data
- Supplementary Data - Supplementary Data
- Supplementary Data - Supplementary Data
- Supplementary Data - Supplementary Data
