# Supplementary material for: 2,4-Dichlorophenoxyacetic acid promotes S-nitrosylation and oxidation of actin affecting cytoskeleton and peroxisomal dynamics
Source: J Exp Bot. 2014 Jun 9;65(17):4783–93. doi: 10.1093/jxb/eru237 (PMC4144765; doi:10.1093/jxb/eru237)
Supplement: Supplementary Data [file supp_eru237_jexbot121160_file001.pdf]

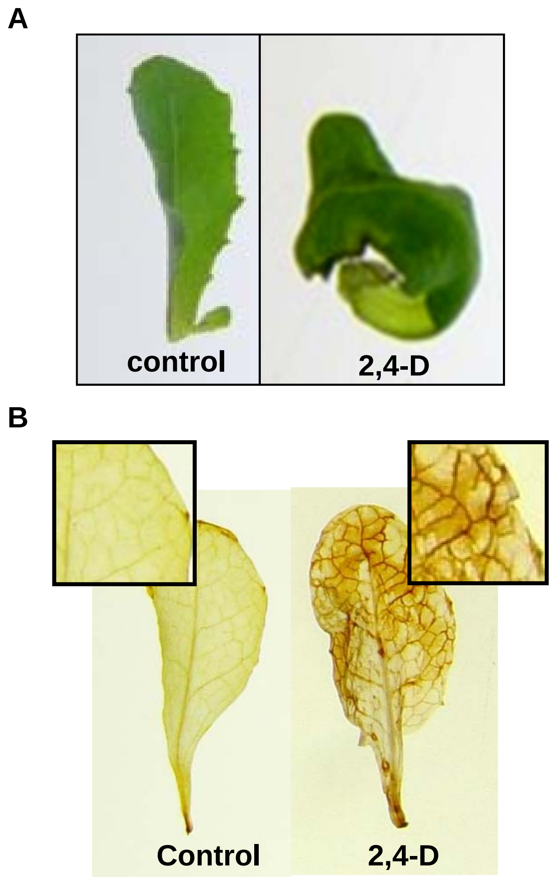

Figure S1: 2,4-D produces epinasty in *Arabidopsis* leaves and accumulation of H_2O_2 mainly in vascular tissue. Leaves were treated with 23 mM 2,4-D, and the effect were analyzed after 72 h of treatment. A) 2,4-D induces leaf epinasty. B) Histochemical analysis of H_2O_2 production was carried out with DAB. Boxes show higher magnification of vascular tissue.

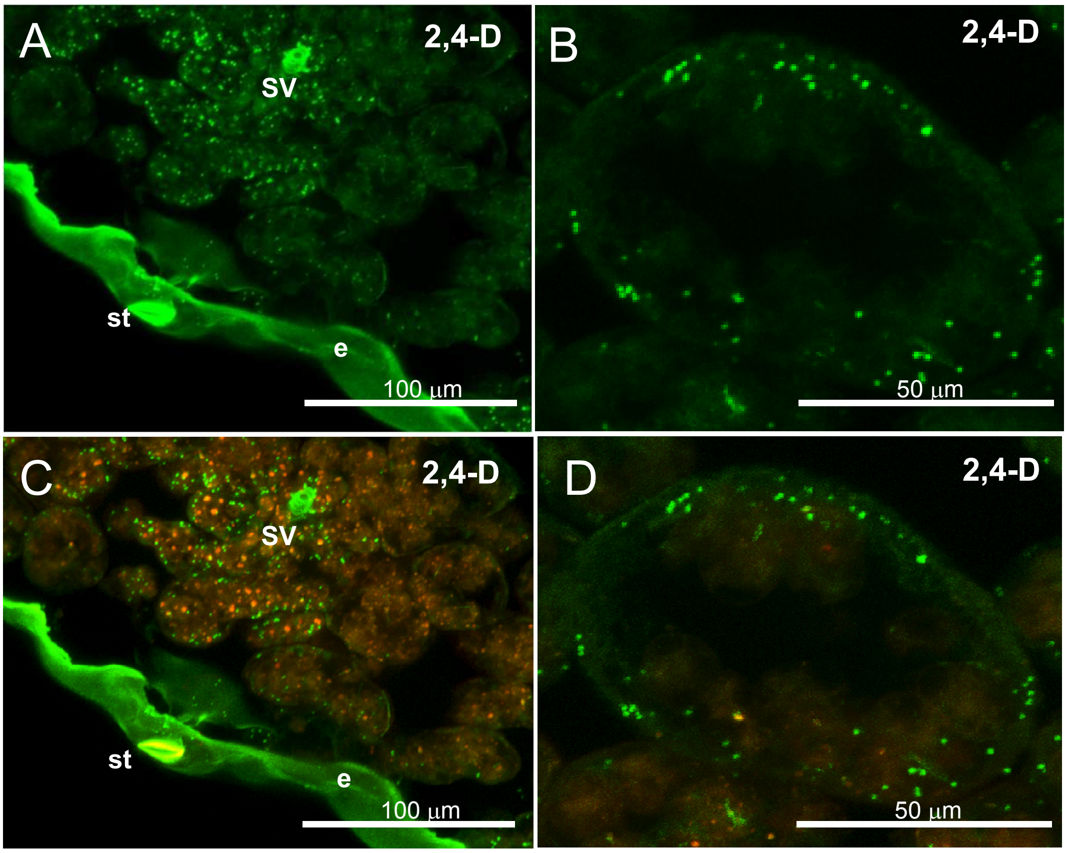

Figure S2. Imaging of $O_2^{\cdot -}$ production by CLSM using DHE (Ex/Em: 450-490/520 nm, green) and chlorophyll autofluorescence (red) showing magnifications of mesophyll cells from 2,4-D treated plants. A) $O_2^{\cdot -}$-dependent DHE fluorescence associated to secondary veins (SV), stomata (st) and epidermis (e). B) High magnification of a mesophyll cell showing DHE associated to small organelles (mitochondria and peroxisomes). C and D show the merge of $O_2^{\cdot -}$-dependent DHE fluorescence (green) and the chlorophyll autofluorescence (red, Ex/Em: 633/680 nm).

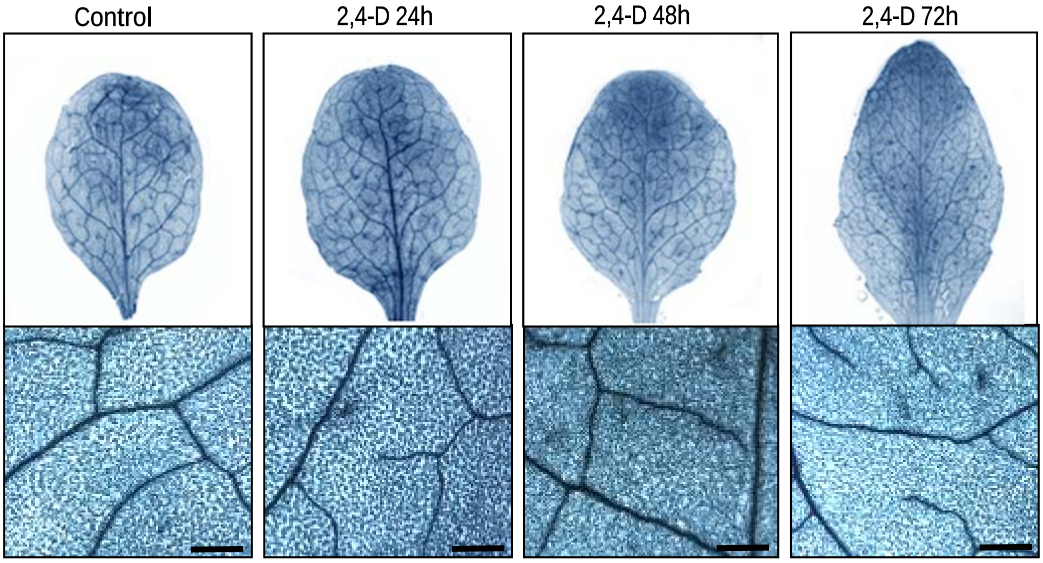

Figure S3. Histochemical staining with Trypan Blue from Arabiodospis leaves treated at different times with 2,4-D (23 mM). Leaves were stained with Trypan Blue as indicated in Materials and Methods. Bars= 200 µm.
