# Supplementary material for: 2,4-Dichlorophenoxyacetic acid promotes S-nitrosylation and oxidation of actin affecting cytoskeleton and peroxisomal dynamics
Source: J Exp Bot. 2014 Jun 9;65(17):4783–93. doi: 10.1093/jxb/eru237 (PMC4144765; doi:10.1093/jxb/eru237)
Supplement: Supplementary Data [file supp_eru237_Supplementary_Material.docx]

**Supplementary Material**

**Video S1A:** Movies showing peroxisomal dynamics in epidermal cells from control *Arabidopsis* plants expressing the GFP-SKL.

**Video S1B :** Movies showing the effect of 23 mM 2,4-D on peroxisomal dynamics in epidermal cells from control *Arabidopsis* plants expressing the GFP24 SKL.

**Video S2A**: Movies showing peroxisomal and mitochondrial dynamics in epidermal cells from control double markers *Arabidopsis* px-ck x mt-yk plants.

**Video S2B**: Movies showing the *effect* of 2,4-D on peroxisomal and mitochondrial dynamics in epidermal cells from double markers *Arabidopsis* px29 ck x mt-yk plants.
